# Dexamethasone and post-dural puncture headache in women who underwent cesarean delivery under spinal anesthesia: A systemic review and meta-analysis of randomized controlled trials

**DOI:** 10.1016/j.amsu.2021.01.024

**Published:** 2021-01-18

**Authors:** Efrem Fenta, Simegnew Kibret, Metages Hunie, Diriba Teshome

**Affiliations:** a Department of Anesthesia, College of Health Sciences, Debre Tabor University, Debre Tabor, Ethiopia

**Keywords:** Dexamethasone, Incidence, Post-dural puncture headache, Severity, CI, Confidence Interval, IV, Intravenous, MA, Meta-analysis, MD, Mean Difference, OR, Odds Ratio, SD, Standard Deviation, SR, Systemic Review, VAS, Visual Analogue Score

## Abstract

**Background:**

Post-dural puncture headache is a common complication after spinal anesthesia for women who undergo cesarean delivery. Intravenous (IV) dexamethasone has been used to reduce the incidence and severity of PDPH with controversial results. This Systemic review and meta-analysis aimed to assess the effects of IV dexamethasone on PDPH.

**Methods:**

This study is reported as per Preferred Reporting Items for Systematic and Meta-analysis. The primary outcome was the incidence and severity of PDPH. The secondary outcome variables were the postoperative total analgesic requirement and incidence of nausea and/or vomiting. Twelve randomized controlled trials with a total of 1548 women were included.

**Results:**

Intravenous (IV) dexamethasone had no effect on the incidence of PDPH (OR = 0.64; CI, 0.39 to 1.05; I^2^ = 71%, P = 0.08). Intravenous dexamethasone did not show a significant difference in the incidence of PDPH at 24 h at 48 h, and within one week postoperatively with p-values of less than 0.05. In a random-effect model, a pooled analysis showed that IV dexamethasone had no effect on the severity of PDPH in VAS (MD = 0.78; CI, −2.27 to 0.71; I^2^ = 98%, P = 0.30).

**Conclusion:**

Intravenous dexamethasone failed to decrease the incidence and severity of PDPH in women who underwent cesarean delivery under spinal anesthesia.

## Highlights

• PDPH is one of the unwanted complications of spinal anesthesia.
• Intravenous dexamethasone failed to decrease the incidence of PDPH.
• Intravenous dexamethasone failed to decrease the severity of PDPH.
• Intravenous dexamethasone failed to decrease the analgesic consumption.

## 1 Introduction

### 1.1 Description of the condition

Spinal anesthesia has been the anesthetic technique of choice for cesarean delivery unless it is contraindicated [[1], [2], [3], [4]]. Post-dural puncture headache (PDPH) is among common spinal anesthesia-related side effects for mothers who underwent cesarean delivery. It might appear several hours to a week after dural puncture, and could be a cause of poor patient outcome [[5], [6], [7], [8]]. (see Table 1)

**Table 1:** Characteristics of included studies.

Author/s, study year, cite	Number of patients (Total, IV Dexa, placebo) and study design	Type of surgery	Type of Anesthesia	Dexamethasone group/time, dose/	Placebo group/time, dose, type/	Outcomes
Doroudian et al., 2011 [34]	Total = 178, Randomized double-blind, Placebo-controlled, clinical trial	Lower extremity orthopedic surgery	Spinal anesthesia.	Received 2 ml/8 mg intravenous (i.v) Dexamethasone After termination of surgery	2 ml of normal saline After termination of surgery	There was no statistically significant difference between groups regarding the Incidence of PDPH. However, the intensity of headache differed between the two groups being less severe if IV dexamethasone had been given prophylactically
Anbarlouei et al., 2020 [35]	ASA I &II women Total = 216, Control = 72, IV Dexa = 72 Hydrocortisone = 72 Clinical trial	Cesarean section	Spinal anesthesia	8 mg dexamethasone IV 200 mg of hydrocortisone	2 ml of normal saline	The incidence of PDPH in the control group, 1 (3.33%), 6 (20%), 11 (36.67%), 12 (40%) of headaches developed immediately, 6, 24, and 48 h after cesarean section respectively. On the other hand, 1 (10%), 5 (50%), and 4 (40%) of headache cases in the hydrocortisone group, and 3 (18.75%), 5 (31.25%), and 8 (50%) of headaches in the dexamethasone group initiated at 6, 24, and 48 h after cesarean section respectively. There were no statistically significant differences among the three groups regarding the incidence of the headache immediately, 6, 24, and 48 h following cesarean section. The prevalence of headache was 41.6% (30 of 72 patients) in the placebo group, 22.2% (16 of 72 patients) in the dexamethasone group, and 13.8% (10 of 72 patients) in the hydrocortisone group Regarding the pain severity, the headaches were significantly more severe in the control group compared with the hydrocortisone and dexamethasone groups at 24 (P = 0.02), and 48 (P = 0.01) hours, and 1 week (P = 0.001) after cesarean section.
Shokrpour et al., 2018 [36]	Total = 120 Control = 40, IV Dexa = 40 Ondansetron = 40 a double-blind clinical trial	mothers candidated for elective, second time cesarean	Spinal anesthesia	8 mg IV Dexamethasone, 8 mg of IV ondansetron.	2 ml of distilled water.	The mean period of hospitalization in days was: 2.1 ± 0.8, 2.01 ± 1.1, 2.2 ± 0.9 in Ondansetron, Dexamethasone, and Placebo with a p-value of 0.63 respectively. The occurrence of the PDPH were: 10%, 7.5%, and 20% in Ondansetron, Dexamethasone, and Placebo with a p-value of 0.001 respectively within 48 h VAS at 12 h 5.01 ± 1.1, 3.6 ± 0.9, and 5.5 ± 1.8 in Ondansetron, Dexamethasone, and Placebo with a p-value of 0.02 respectively VAS at 24 h 5.03 ± 1.4, 4.6 ± 1.7, and 5.8 ± 2.1in Ondansetron, Dexamethasone and Placebo with a p-value of 0.03 respectively VAS at 48 h 2.01 ± 0.7, 1.01 ± 0.6, and 2.9 ± 1.1in Ondansetron, Dexamethasone, and Placebo with a p-value of 0.03 respectively. The average analgesic used to treat headache within 48 h (mg) were: 112.5 ± 7.6, 100.8 ± 8.2, and 150.7 ± 9.1 in Ondansetron, Dexamethasone, and Placebo with a p-value of 0.01 respectively.
Yousefian et al., 2017 [37]	Total = 150, Control = 50, IV Dexa = 50, IV ondasetron = 50 A double-blind clinical trial	Cesarean section	Regional anesthesia	8 mg dexamethasone IV, 4 mg of ondansetron	2 ml of Normal saline	The prevalence of headache was 9(18%), 0(0%), and 0(0%) in placebo, ondansetron, and dexamethasone groups respectively with a p-value of <0.05 within 48 h. The prevalence of Nausea and vomiting during and after surgery were 15(30%), 0(0%), and 0(0%) in placebo, ondansetron, and dexamethasone groups respectively with a p-value of <0.05 within 48 h.
Hamzei et al., 2012 [38]	Total = 160 Control = 80, IV Dexa = 80, A single-blind randomized, control trial	Cesarean section	Spinal anesthesia	8 mg IV dexamethasone	control	The incidence of headache in the first 24 h were: 2 (2.5), and 10 (12.5%) in dexamethasone and control groups respectively. Incidence of headache in the week were: 9 (11.3%), and 26 (32.5%) in dexamethasone and control groups respectively. The severity of headache in the first 24 h in VAS were: 2.5 ± 2.12, and 2.6 ± 2.55 in dexamethasone and control groups respectively. The severity of headache in the first week in VAS were: 4.66 ± 2.82, and 4.7 ± 2.75 in dexamethasone and control groups respectively.
Yousefshahi et al., 2012 [39]	Total = 360 IV dexa = 182 placebo = 178 aged 18–44 years, A prospective Double blind randomized placebo-controlled study	Cesarean section	Spinal anesthesia	2 ml/8 mg IV dexamethasone	(2 ml 0f normal saline) intravenously	Incidence of intraoperative nausea and vomiting were: 17 (44.7%), and 21 (48.8%) in placebo and dexamethasone respectively. Over the Incidence of PDPH were: 11(6.2%), and 28 (15.4%) in placebo, and dexamethasone groups respectively Incidence of PDPH within first 24 h were: 8, and 24 in placebo, and dexamethasone groups respectively. Incidence of PDPH within the second 24 h was: 6, and 7 in placebo, and dexamethasone groups respectively with a p-value of 0.046. The severity of PDPH with the first 24 h in VAS were: 2.375, and 2.624 in placebo, and dexamethasone groups respectively with a p-value of 0.678. The severity of PDPH with the second 24 h in VAS were: 1.83, and 2.0 in placebo, and dexamethasone groups respectively
Shakhsemampour et al., 2018 [40]	Total = 104, Control = 52, IV Dexa = 52, aged 15–45, ASA I–II randomized double-blind clinical study	Elective cesarean section	Spinal anesthesia	2 ml/8 mg of dexamethasone IV	2 ml of normal saline.	Incidence of PDPH in recovery was 5, and 3 in placebo, and dexamethasone groups respectively with a p-value of 0.715. Incidence of PDPH within 48 h was 10, and 8 in placebo, and dexamethasone groups respectively with a p-value of 0.604. The severity of headache in VAS (M±SD) at recovery were 0.75 ± 1.19, and 0.73 ± 1.64 in dexamethasone, and placebo groups respectively with a p-value of 0.943. The severity of headache in VAS (M±SD) within 48 h was 1.05 ± 2.32, and 1.01 ± 2.31in dexamethasone, and placebo groups respectively with a p-value of 0.93
Okpala et al., 2020 [41]	Total = 192, Control = 96, IV Dexa = 96, A double blind placebo controlled randomized trial	Cesarean section	spinal anesthesia	2 ml/8 mg of dexamethasone.	2 ml normal saline IV.	The incidence of PDPH was 8 (8.3%) vs 24 (25.0%); in dexamethasone, and control groups with first 4 days respectively with a p-value of 0.002, and The incidence of PDPH was 7 (7.29%) vs 16 (16.67%); in dexamethasone, and control groups with first 24 h respectively while the incidence of nausea was 11.5% vs 25.0% in dexamethasone, and control groups in the first four days respectively with a p-value of 0.015. The severity of headache means VAS rank 110.25 versus 82.75 on the first day, and 10.25 versus 90.75 within 4 days for control and IV dexamethasone groups respectively.
Najafi et al., 2014 [42]	Total = 268, Control = 134, IV Dexa = 134, aged 18–40 yrs Clinical trial	Any surgery with spinal anesthesia	Spinal anesthesia	2 ml of dexamethasone (8 mg IV) injected into the patient's epidural space	2 ml of normal saline was injected into the patient's epidural space	The overall prevalence of headache at any time within the 1st week following the surgical procedure was five cases (3.7%) and 11 cases (8.2%) in the control and case groups, respectively with a p-value of 0.122. The prevalence of headache within 24 h was 3 (2.2%), and 5 (3.7%) in the control, and dexamethasone groups respectively with a p-value of 0.722. The prevalence of headache within 72 h was 5 (3.7%), and 8 (6.0%) in the control, and dexamethasone groups respectively with a p-value of 0.571. The prevalence of headache within 7 days was 2 (1.5%), and 2 (1.5%) in the control, and dexamethasone group respectively
Motaghi et al., 2011 [43]	Total = 60 Control = 30, IV Dexa = 30, aged 18–45 years, ASA = I and II A prospective, randomized, placebo-controlled	Elective Cesarean section	Spinal anesthesia	8 mg of IV Dexamethasone	2 ml of intravenous normal saline	8 mg of intravenous Dexamethasone does not have any significant effect on headache prevalence in parturients after spinal anesthesia for elective cesarean section.
Tavakol K et al., 2007 [44]	Total = 35, Control = 35, IV Dexa = 35, aged 21–44 yrs, ASA I-III A randomized clinical trial	Cesarean section	Spinal anesthesia	IV drip of dexamethasone 0.2 mg/kg (maximum 16 mg) in 1 L of normal saline for 2 h	Control	Results showed that mean of VAS pain score before treatment was 6.5 ± 1.8 and decreased to 1.6 ± 1.2 after treatment indicating a decrease of 77% in pain among the subjects
Naghibi et al., 2014 [45]	Total = 140, IV dexa = 35, IV dexa and amino = 35, IV amino = 35 Placebo = 35 aged 20–65 years, randomized, double-blind, placebocontrolled study	lower extremity surgery	Spinal anesthesia	5 ml of (0.1 mg/Kg dexamethasone IV), 5 ml of (1.5 mg/Kg aminophylline), 5 ml of (0.1 mg/Kg dexamethasone + 1.5 mg/Kg aminophylline)	5 ml normal saline	Patients in group aminophylline plus dexamethasone had a significantly lower incidence of PDPH 5.88% vs. 20.58% for group aminophylline, 17.14% for group Dexamethasone, and 42.8% with P-value of <0.05. Patients in group aminophylline plus dexamethasone require less analgesia compared with groups aminophylline, Dexamethasone, and Placebo throughout 6–48 h 1.2 ± 0.4 vs. 2.3 ± 0.75 for group Aminophylline, 1.8 ± 0.6 for group Dexamethasone, and 3.3 ± 1 for placebo group with P-value of <0.05. postoperative analgesia acetaminophen iv in mg 1.2 ± 0.4 for aminophylline plus dexamethasone group, 2.3 ± 0.75 for aminophylline group, 1.8 ± 0.6 for dexamethasone group, and 3.3 ± 1 for placebo group

Dural puncture and subsequent cerebrospinal fluid (CSF) leakage is the most accepted mechanism for the induction of headache [[9], [10], [11], [12]]. CSF leakage through the dural hole and reduction in CSF pressure lessens the cushioning effect of the brain, allowing it to sag within the intracranial vault and stimulation of dural pain receptors especially in an upright position [[13], [14], [15]].

There were different techniques to prevent and treat PDPH like bed rest, hydration, non-opioid analgesics, caffeine, codeine, and steroids [[16], [17], [18], [19]]. Different studies tried to show the effects of intravenous dexamethasone and found controversial results. Therefore, this study aims to find the pooled effects of intravenous dexamethasone on PDPH.

### 1.2 How the intervention might work

The exact mechanism of action of how dexamethasone helps in reducing the incidence and severity of PDPH and pain is not well established. Intravenous dexamethasone might reduce PDPH and pain by inhibiting the inflammatory process which is important in the pain cascading pathway [[20], [21], [22], [23], [24], [25]].

### 1.3 Why it is important to do this SR and MA

There were controversial results regarding the effect of intravenous dexamethasone on reducing the occurrence and severity of PDPH, which necessitates this SR and MA. Some studies showed that dexamethasone increased significantly the frequency and severity of PDPH after cesarean delivery [26,27], other studies dexamethasone fail to reduce the incidence and severity of PDPH in different dosage [28,29], while other studies show that steroids decrease its incidence and severity [1,30,31]. There were SR and MA regarding the effects of IV dexamethasone on the incidence and severity of PDPH for women undergoing cesarean delivery under regional anesthesia.

This SR and MA address the effectiveness of IV dexamethasone on PDPH occurrence and severity, and it might be supportive evidence for the scientific world. This Systemic review and meta-analysis aimed to assess the effects of IV dexamethasone administration on PDPH occurrence and severity.

## 2 Methods

### 2.1 Criteria for considering studies for this review

This study is reported as per Preferred Reporting Items for Systematic and Meta-analysis [32] and it is a high-quality systemic review based on AMSTAR 2 checklist self-evaluation [33]. Twelve randomized controlled trials with a total of 1548 patients were included. This SR and MA is registered in research registry with registration number reviewregistry1063 available at https://www.researchregistry.com/browse-the-registry#registryofsystematicreviewsmeta-analyses/registryofsystematicreviewsmeta-analysesdetails/5ff9a85073f73d001b5b2283/

### 2.2 Types of studies

Relevant articles were identified by four authors through their titles and abstracts from databases (Medline, Cochrane library, and Google scholar) and hand search. The clinical trials, free full texts, and human species were included.

### 2.3 Types of participants

The participants included in this SR and MA were women who underwent cesarean delivery under Spinal anesthesia.

### 2.4 Types of interventions

Intravenous dexamethasone is the intervention group in this SR and MA while normal saline is considered as a placebo group.

### 2.5 Types of outcome measures

The primary outcome in this SR and MA were the incidence of PDPH and severity of PDPH in VAS while the secondary outcomes were a total postoperative analgesic requirement and the incidence of postoperative nausea and/or vomiting.

### 2.6 Search methods for identification of studies

#### 2.6.1 Electronic searches

The MEDLINE, Cochrane library, and google scholar from inception to October 2020, were searched for clinical trials comparing the effectiveness of intravenous dexamethasone versus placebo on the effect of PDPH.

We searched the following databases for the literature of the English language by using the following terms: (Headache, Post-Dural Puncture OR Headaches, Post-Dural Puncture OR Post Dural Puncture Headache OR Post-Dural Puncture Headaches OR Postdural Puncture Headache OR Headache, Postdural Puncture OR Headaches, Postdural Puncture OR Postdural Puncture Headaches OR Post-Lumbar Puncture Headache) AND (Dexamethasone OR Glucocorticoids OR Steroids). The included articles were limited to ‘Clinical trials’ and human studies.

### 2.7 Searching other sources

The hand search was applied to studies to identify additional literature by using key terms and via cross-references, links, and citations in google scholar and PubMed.

### 2.8 Data collection and analysis

#### 2.8.1 Exclusion criteria

Studies that compared IV dexamethasone with other interventions of PDPH without a control group, IV dexamethasone without spinal anesthesia, IV dexamethasone with other additives.

### 2.9 Data extraction and management

Authors’ names with a year of publication, study characteristics, type of surgery, type of anesthesia, a dose of dexamethasone, normal saline dose, and outcomes were extracted.

The titles and abstracts of all references identified in the searches were reviewed by four authors. Studies that are not met inclusion criteria were excluded. Full paper copies of included studies will be reviewed by four authors independently, and decisions made regarding selection/rejection. The disagreements arising were resolved by the discussion of all the reviewers.

### 2.10 Assessment of risk of bias in included studies

The risk of bias was assessed by using the Cochrane risk of bias tool and noted as being low, unclear, or high risk by the four researchers independently. Trials were rated according to random sequence generation (selection bias), allocation concealment (selection bias), blinding of participants and personnel (performance bias), blinding of outcome assessment (detection bias), incomplete outcome data (attrition bias), selective reporting (reporting bias), and other bias. The disagreements arising were considered and resolved by discussion. Concerning incomplete outcome data, studies were classified as low risk of bias if the follow-up rate was ≥80%. For selective outcome reporting bias, studies were classified as low risk of bias if trials were preregistered and their protocols were available for full review [32].

### 2.11 Statistical analysis

Analyses were done with Review Manager version 5.4.1; Cochrane Library, Oxford, UK. Dichotomous variables (incidence of PDPH and incidence of PONV), and continuous variables (severity of PDPH in VAS and analgesic requirement in mg) were reported as odds ratio and mean differences with 95% CIs respectively. The *I*^2^ test was used to assess the Heterogeneity of the outcomes. A fixed-effect model and a random-effect model were used when *I*^2^<50% and *I*^2^>50% respectively. The funnel-plot analysis was used to assess potential publication biases [46].

## 3 Results

### 3.1 Description of studies

**Fig. 1:**
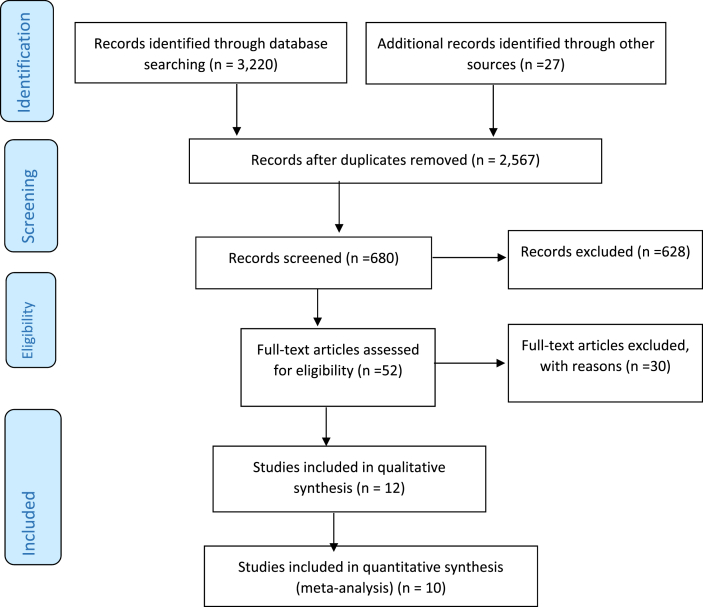
PRISMA flow chart of literature search results.

The primary literature search initially identified 3247 articles. After duplicates were removed 2567 were left for further screening by abstract and title which gave us 52 full text available clinical trials of human studies for inclusion. Then 12 studies were used for qualitative (SR) while 10 studies were used for quantitative (MA) (Fig. 1).

**Fig. 2:**
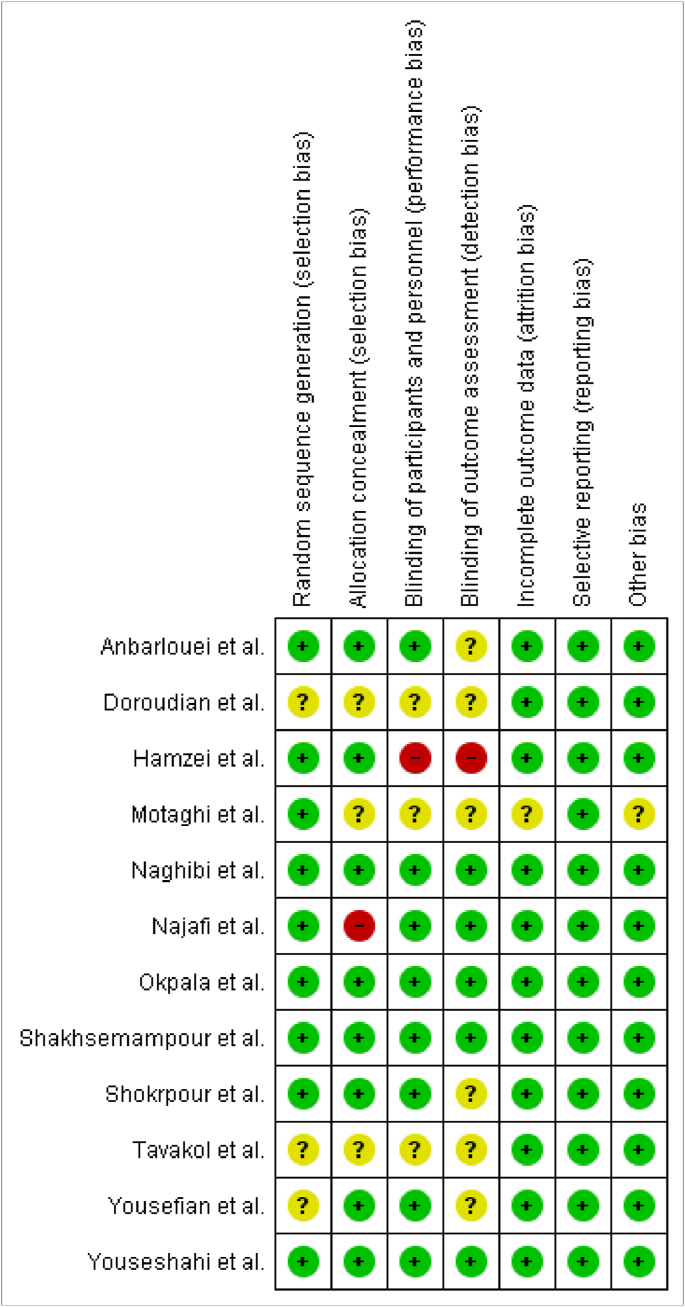
Risk of bias assessment.

The risks of bias for included studies were evaluated by four authors and discussed as low risk, high risk, and unclear risk. Hamzei et al. [38] high risk of bias in blinding of participants and personnel, and blinding of outcome assessment. Motaghi et al. [43] low risk of bias in random sequence generation, and reporting bias, while the unclear risk of bias in allocation concealment, blinding of participants and personnel, and blinding of outcome assessment and in other biases (Fig. 2).

### 3.2 Assessment of publication bias

A funnel plot was created for the primary outcome and visually inspected to assess publication bias. A symmetrical inverted funnel plot shows no publication bias.

### 3.3 Incidence of post-dural puncture headache

**Fig. 3:**
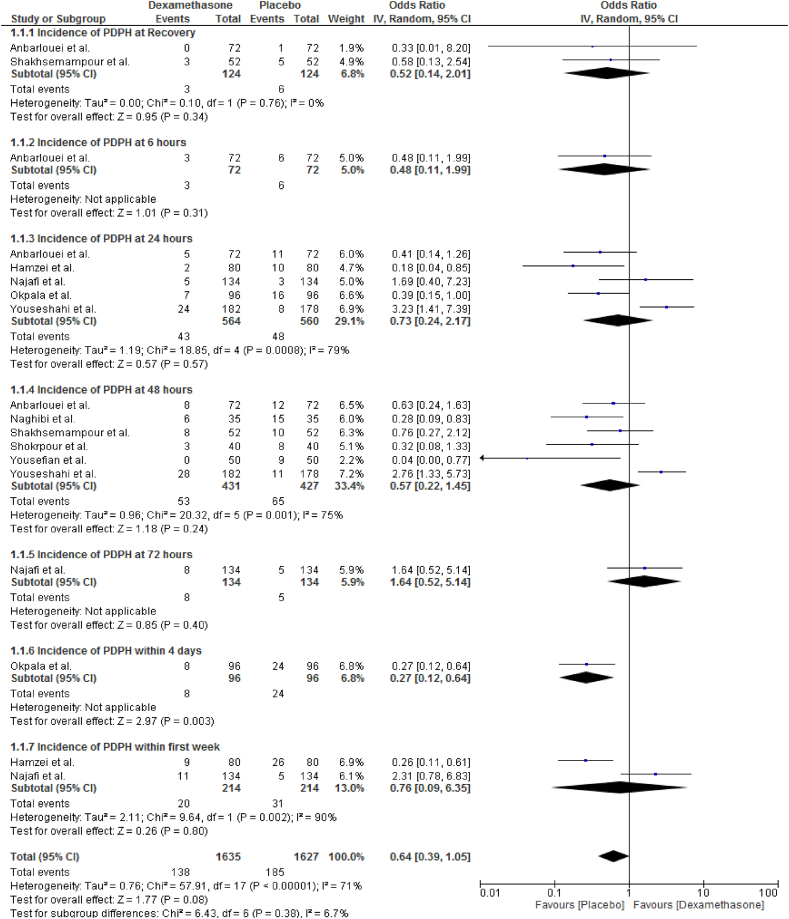
Effects of intravenous dexamethasone on the incidence of PDPH.

The incidence of PDPH was reported by nine RCTs [[35], [36], [37], [38], [39],^41^,^42^,^45^]. In a random-effect model, a pooled analysis of nine clinical trials, showed that intravenous dexamethasone has no statistically significant effect on the frequency of PDPH (OR = 0.64; CI, 0.39 to 1.05; I^2^ = 71%, P = 0.08). Intravenous dexamethasone did not show a significant difference in incidence of PDPH at 24 h [35,38,39,41,42] (OR = 0.73; CI, 0.24 to 2.17; I^2^ = 79%, P = 0.57), at 48 h [[35], [36], [37],^39^,^40^,^45^] (OR = 0.57; CI, 0.22 to 1.45; I^2^ = 75%, P = 0.24), and within one week postoperatively [38,42] (OR = 0.76; CI, 0.09 to 6.35; I^2^ = 90%, P = 0.80) (Fig. 3).

The severity of Post-dural puncture headache.

**Fig. 4:**
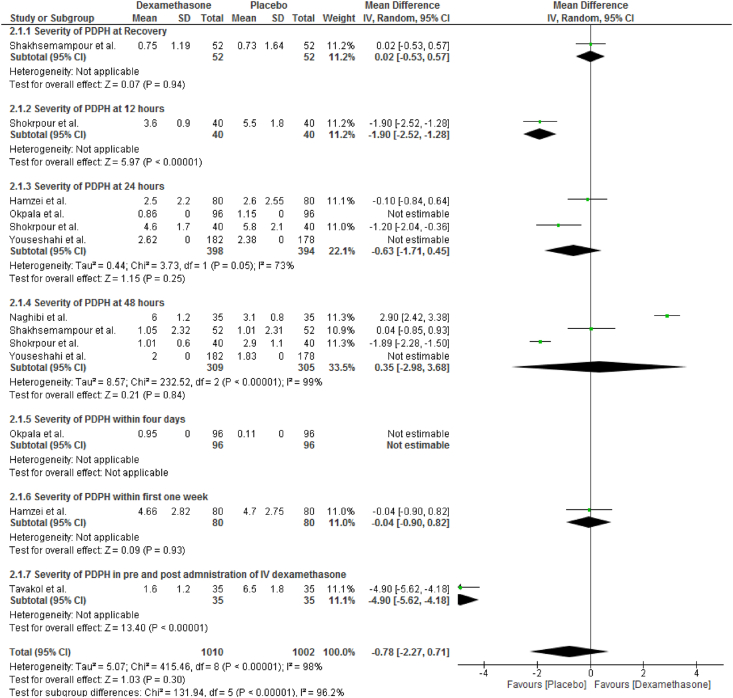
Effects of intravenous dexamethasone on the severity of PDPH in VAS.

The severity of PDPH was reported by 6 RCTs [36,[38], [39], [40], [41],^45^]. In a random-effect model, a pooled analysis of six clinical trials showed intravenously dexamethasone had no a statistically significantly difference between groups on severity of PDPH in VAS (MD = 0.78; CI, −2.27 to 0.71; I^2^ = 98%, P = 0.30). Intravenous dexamethasone did not show a statistical significant difference in severity of PDPH in VAS at 24 h [36,38,39,41] (MD = −0.63; CI, −1.71 to 0.45; I^2^ = 73%, P = 0.25), and at 48 h [36,39,40,45] (MD = 0.35; CI, −2.98 to 3.68; I^2^ = 99%, P = 0.84) (Fig. 4).

### 3.4 Analgesic requirement

**Fig. 5:**
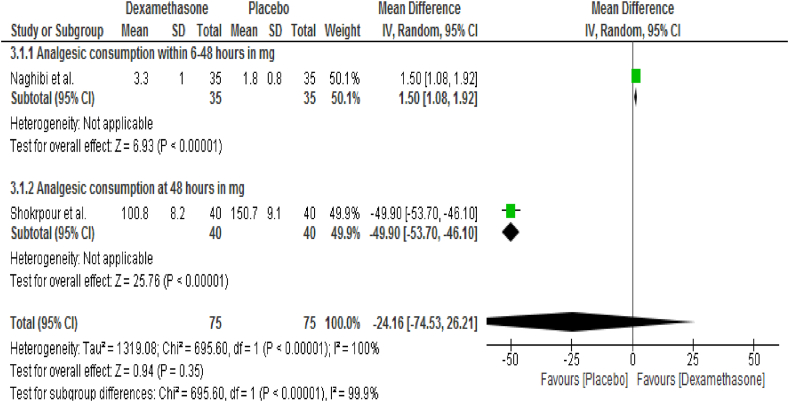
Effects of intravenous dexamethasone on the total postoperative analgesic consumption.

Two studies [36,45] reported the analgesic consumption. Intravenous dexamethasone failed to show a statistical significant difference in total postoperative analgesic consumption within 48 h (MD = −24.16; CI, −74.53 to 26.21; I^2^ = 100%, P = 0.35) (Fig. 5).

### 3.5 Incidence of nausea and/or vomiting

**Fig. 6:**
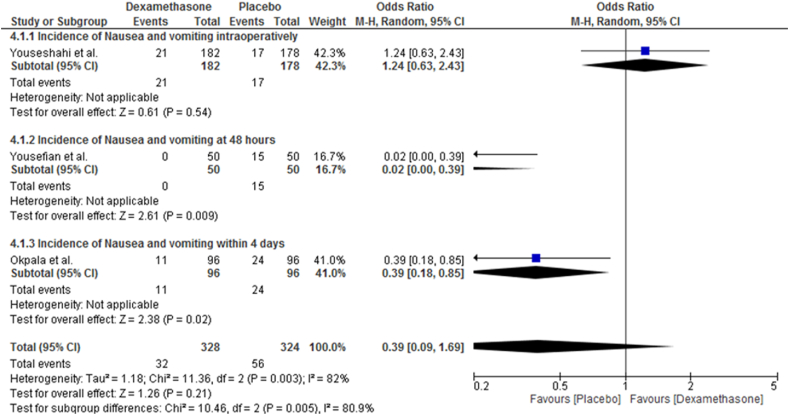
Effects of intravenous dexamethasone on the incidence of postoperative nausea and/or vomiting.

The incidence of PDPH was reported by three RCTs [37,39,41]. In a random-effect model, a pooled analysis showed that intravenous dexamethasone has no statistically significant effect on the prevalence of nausea and/or vomiting (OR = 0.39; CI, 0.09 to 1.69; I^2^ = 82%, P = 0.21) (Fig. 6).

## 4 Discussion

Intravenous dexamethasone might reduce the incidence and severity of PDPH and pain through glucocorticoid steroid receptors that cause vasoconstriction and reduce the absorption of administered local anesthetic, inhibiting the production of inflammatory mediators [21,25]. There were controversial results regarding the effect of intravenous dexamethasone on reducing the occurrence and severity of PDPH, which necessitates this SR and MA.

The results of our systemic review and meta-analysis revealed that intravenous dexamethasone failed to decrease the occurrence and the severity of PDPH in women who underwent cesarean delivery under spinal anesthesia. Contrary to this SR and MA researches done by Ona et al., Yousefshahi et al., showed that IV dexamethasone increases the occurrence of PDPH [26,27]. In agreement with this SR and MA studies done by Yang et al., and Mahmoud et al. found that the use of IV dexamethasone has no statistically significant benefit to the occurrence and severity of PDPH [28,29], while some studies showed that steroids decrease its incidence and severity [1,30,31]. There were SR and MA regarding the effects of IV dexamethasone on the incidence and severity of PDPH for women undergoing cesarean delivery under regional anesthesia.

This SR and MA address the effectiveness of IV dexamethasone on PDPH occurrence and severity, and it might be supportive evidence for the scientific world. This Systemic review and meta-analysis aimed to assess the effects of IV dexamethasone administration on PDPH occurrence and severity.

## 5 Conclusions

Intravenous dexamethasone failed to decrease the incidence and severity of PDPH in women who underwent cesarean delivery under spinal anesthesia.

### Limits and challenges

During this study, we have encountered the difficulty of found freely available studies and we tried to search them by using different databases.

## Availability of data and materials

All data generated or analyzed during this study are available in this manuscript.

## Provence and peer-reviewed

Not commissioned, externally peer-reviewed.

## Competing interests

The authors declare that they have no competing interests.

## Authors' contributions

Alemnew EF, Wubetu SK, Belay MH, and Lemma DT performed the literature search, assessment of articles, data extraction, statistical analysis, manuscript preparation.

## Ethics approval and consent to participate

Not applicable.

## Funding

No.
